# *PLOS Neglected Tropical Diseases* broadens its coverage of envenomings caused by animal bites and stings

**DOI:** 10.1371/journal.pntd.0009481

**Published:** 2021-06-17

**Authors:** José María Gutiérrez, Jean Philippe Chippaux, Geoffrey K. Isbister

**Affiliations:** 1 Instituto Clodomiro Picado, Universidad de Costa Rica, San José, Costa Rica; 2 Université de Paris, MERIT, IRD, Paris, France; 3 CRT, Institut Pasteur, Paris, France; 4 Clinical Toxicology Research Group, University of Newcastle, Newcastle, New South Wales, Australia; George Washington University School of Medicine and Health Sciences, UNITED STATES

Envenomings from animal bites and stings are particularly frequent in developing countries where they dramatically affect the most deprived populations. A wide variety of animals from different taxa synthesize and inject venoms either as a trophic mechanism to subdue and digest prey or as a defense against predators and other enemies. These toxic secretions can also be injected into humans or domestic animals and significantly impact human and veterinary health. Bites by venomous snakes cause 1.8 to 2.7 million cases of envenomings every year, with 81,000 to 138,000 fatalities, and, possibly, leaving more people with permanent physical and psychological sequelae [1]. This burden of illness largely occurs in impoverished rural populations in sub-Saharan Africa, Asia, and Latin America. In addition, snakebite has a heavy socioeconomic impact, because it predominantly affects the young working population in low- and middle-income countries [2]. For these reasons, snakebite envenoming is included in the list of neglected tropical diseases of the World Health Organization.

As one of the foremost journals in tropical medicine and neglected tropical diseases, *PLOS Neglected Tropical Diseases* has regularly published contributions on various aspects of snakebite and snake envenoming for over a decade. However, envenoming by animal bites and stings goes beyond snakebite and includes scorpions and spiders, insects, jellyfish, and other marine creatures.

Scorpion stings cause severe and potentially fatal envenoming that mainly affects children in regions of Africa, Asia, and Latin America [3]. Scorpion sting envenoming shares many of the features that characterize neglected tropical diseases, because it disproportionately affects families in resource-poor regions of the world, which usually lack access to medical facilities.

Spider bite is also a significant problem in many parts of the world, with bites from widow spiders causing a painful neurotoxic envenoming syndrome (latrodectism) that occurs worldwide and cutaneous and systemic loxoscelism that can result in necrotic ulcers and haemolytic and renal complications [4]. Other arthropods can also inflict systemic envenoming, ranging from caterpillar envenoming in South America, massive bee attacks associated with potentially fatal multi-organ injury, and tick envenoming that can result in paralysis. Marine envenoming is rarely fatal, but jellyfish stings cause thousands of cases each year in many parts of the world.

To broaden the coverage of a variety of conditions that share the features of currently recognized neglected tropical diseases, *PLOS Neglected Tropical Diseases* will accept manuscripts dealing with scorpion sting envenoming, in addition to snakebite envenoming. Moreover, it will consider manuscripts on envenomings by other animals, especially if the studies deal with epidemiological, clinical, therapeutic, social science, and/or public policy aspects. Studies on these topics submitted to *PLOS Neglected Tropical Diseases* must be pertinent to public health in resource-poor settings. The reports on local and original initiatives on these issues in developing countries will be particularly appreciated. It is expected that such broadening in the thematic spectrum of diseases associated with envenomings by animal bites and stings will contribute to the recognition of the public health relevance of these conditions and will foster the search for solutions to reduce their impact on human welfare.
